# Supplementary material for: A comprehensive economic assessment of the burden of obesity in Kuwait
Source: PLoS One. 2026 Mar 4;21(3):e0344040. doi: 10.1371/journal.pone.0344040 (PMC12959657; doi:10.1371/journal.pone.0344040)
Supplement: S1 Table — (DOCX) [file pone.0344040.s001.docx]

**A comprehensive economic assessment of the burden of obesity in Kuwait**

**Supporting Information**

**S1** **Table.** **Sources of non-cost data**

| **Parameter** | **Source** | **Ref** |
| --- | --- | --- |
| **Prevalence of obesity** | WHO report | [1] |
| **List of obesity-associated diseases** | Literature | [2] |
| **Number of people living with obesity** | World Obesity Atlas | [3] |
| **Number of morbidity and mortality cases for obesity-associated diseases** | Global Burden of Disease | [4] |
| **Annual number of bariatric surgeries** | Literature | [5] |
| **Average annual number of workdays lost** | Literature | [6] |
| **Average annual number of hospital visits** | Literature | [6] |
| **Average annual number of hospital admissions** | Literature | [6] |
| **Life expectancy at time of death** | WHO life tables | [7] |
| **National GDP** | World Bank database | [8] |
| **GDP per capita and its average growth rate** | World Bank database | [8] |
| **Currency exchange rate** | International Monetary Fund and Oanda Corporation | [9, 10] |
| **Budget of the Ministry of Health** | Ministry of Finance, Kuwait | [11] |
| **Healthcare expenditure per capita** | World Bank database | [12] |

*WHO: World Health Organization; GDP: gross domestic product*

**References**

1. WHO. Prevalence of obesity among adults, BMI ≥ 30, age-standardized Estimates by country. Global Health Observatory data repository. 2022 [updated 29 February 2024; cited 2024 8 December]. Available from: <https://www.who.int/data/gho/data/indicators/indicator-details/GHO/prevalence-of-obesity-among-adults-bmi-=-30-(age-standardized-estimate)-(->).

2. Zhou XD, Chen QF, Yang W, Zuluaga M, Targher G, Byrne CD, et al. Burden of disease attributable to high body mass index: an analysis of data from the Global Burden of Disease Study 2021. EClinicalMedicine. 2024;76:102848. Epub 20240924. doi: 10.1016/j.eclinm.2024.102848. PubMed PMID: 39386160; PubMed Central PMCID: PMCPMC11462227.

3. Lobstein T, Brinsden H, Neveux M. World Obesity Atlas 2022 2022 [updated 30 August 2022; cited 2022 30 August]. Available from: <https://www.worldobesityday.org/assets/downloads/World_Obesity_Atlas_2022_WEB.pdf>.

4. Global Burden of Disease Collaborative Network.

Global Burden of Disease Study 2021 (GBD 2021) Results.

Seattle, United States: Institute for Health Metrics and Evaluation (IHME). 2022 [cited 2024 12 October]. Available from: <https://vizhub.healthdata.org/gbd-results/>.

5. Al-Sabah S, Haddad E. The Utilization of Bariatric Surgery in Patients with and without Diabetes: Results from the Second Kuwait National Bariatric Surgery Database Report2022.

6. Nagi MA, Almalki ZS, Thavorncharoensap M, Sangroongruangsri S, Turongkaravee S, Chaikledkaew U, et al. The Burden of Obesity in Saudi Arabia: A Real-World Cost-of-Illness Study. Clinicoecon Outcomes Res. 2025;17:233-46. Epub 20250321. doi: 10.2147/ceor.S504462. PubMed PMID: 40135110; PubMed Central PMCID: PMCPMC11934871.

7. WHO. Global Health Estimates: Life expectancy and leading causes of death and disability. World Health Organization. Department of data and analytics. Geneva, Switzerland 2023 [cited 2023 16 September]. Available from: <https://www.who.int/data/gho/data/themes/mortality-and-global-health-estimates>.

8. World Bank. National accounts data. GDP growth (annual %) - Kuwait 2025 [cited 2024 3 December]. Available from: <https://data.worldbank.org/indicator/NY.GDP.PCAP.KD.ZG?end=2024&locations=KW&start=2005>.

9. International Monetary Fund (IMF). World Economic Outlook Databases. 2024 [updated October 2024; cited 2025 28 June]. Available from: <https://www.imf.org/en/Publications/WEO/weo-database/2024/October>.

10. Oanda Corporation. Forex data services. Currency Converter. Foreign echange rates 2025 [cited 2025 24 February]. Available from: <https://www.oanda.com/currency-converter/en/>.

11. Ministry of Finance, Kuwait. The General Budget for the Fiscal Year 2023/2024 2024 [cited 2025 28 July]. Available from: <https://www.mof.gov.kw/mofbudget/PDF/Budget24-23Eng.pdf>.

12. World Bank. Current health expenditure per capita (current US$), Kuwait 2024 [cited 2025 28 July]. Available from: <https://data.worldbank.org/indicator/SH.XPD.CHEX.PC.CD?locations=KW>.

13. National Cancer Institute. Cancer Trends Progress Report. Economic burden of cancer. 2020 [cited 2025 23 April]. Available from: <https://progressreport.cancer.gov/after/economic_burden>.

14. Casamayor M, Morlock R, Maeda H, Ajani J. Targeted literature review of the global burden of gastric cancer. Ecancermedicalscience. 2018;12:883. Epub 20181126. doi: 10.3332/ecancer.2018.883. PubMed PMID: 30679950; PubMed Central PMCID: PMCPMC6345079.

15. Balkhi B, Alghamdi A, Alqahtani S, Al Najjar M, Al Harbi A, Bin Traiki T. Colorectal cancer-related resource utilization and healthcare costs in Saudi Arabia. Saudi Pharm J. 2023;31(11):101822. Epub 20231012. doi: 10.1016/j.jsps.2023.101822. PubMed PMID: 38023384; PubMed Central PMCID: PMCPMC10630777.

16. Yuen SC, Amaefule AQ, Kim HH, Owoo BV, Gorman EF, Mattingly TJ, 2nd. A Systematic Review of Cost-Effectiveness Analyses for Hepatocellular Carcinoma Treatment. Pharmacoecon Open. 2022;6(1):9-19. Epub 20210824. doi: 10.1007/s41669-021-00298-z. PubMed PMID: 34427897; PubMed Central PMCID: PMCPMC8807829.

17. Hernandez D, Wagner F, Hernandez-Villafuerte K, Schlander M. Economic Burden of Pancreatic Cancer in Europe: a Literature Review. J Gastrointest Cancer. 2023;54(2):391-407. Epub 20220426. doi: 10.1007/s12029-022-00821-3. PubMed PMID: 35474568; PubMed Central PMCID: PMCPMC10435615.

18. Alghamdi A, Alqahtani S. Direct Medical Cost of Breast Cancer in Saudi Arabia. Value in Health. 2020;23:S435. doi: 10.1016/j.jval.2020.08.212.

19. Delgado-Ortega L, González-Domínguez A, Borrás JM, Oliva-Moreno J, González-Haba E, Menjón S, et al. The economic burden of disease of epithelial ovarian cancer in Spain: the OvarCost study. Eur J Health Econ. 2019;20(1):135-47. Epub 20180619. doi: 10.1007/s10198-018-0986-y. PubMed PMID: 29922900; PubMed Central PMCID: PMCPMC6394604.

20. Stucki M, Dosch S, Gnädinger M, Graber SM, Huber CA, Lenzin G, et al. Real-world treatment patterns and medical costs of prostate cancer patients in Switzerland - A claims data analysis. Eur J Cancer. 2024;204:114072. Epub 20240424. doi: 10.1016/j.ejca.2024.114072. PubMed PMID: 38678761.

21. Charbonnel B, Simon D, Dallongeville J, Bureau I, Dejager S, Levy-Bachelot L, et al. Direct Medical Costs of Type 2 Diabetes in France: An Insurance Claims Database Analysis. Pharmacoecon Open. 2018;2(2):209-19. doi: 10.1007/s41669-017-0050-3. PubMed PMID: 29623622; PubMed Central PMCID: PMCPMC5972121.

22. Rittiphairoj T, Bulstra C, Ruampatana C, Stavridou M, Grewal S, Reddy CL, et al. The economic burden of ischaemic heart diseases on health systems: a systematic review. BMJ Glob Health. 2025;10(2). Epub 20250212. doi: 10.1136/bmjgh-2024-015043. PubMed PMID: 39939107; PubMed Central PMCID: PMCPMC11822391.

23. Strilciuc S, Grad DA, Radu C, Chira D, Stan A, Ungureanu M, et al. The economic burden of stroke: a systematic review of cost of illness studies. J Med Life. 2021;14(5):606-19. doi: 10.25122/jml-2021-0361. PubMed PMID: 35027963; PubMed Central PMCID: PMCPMC8742896.

24. Buja A, Rebba V, Montecchio L, Renzo G, Baldo V, Cocchio S, et al. The Cost of Atrial Fibrillation: A Systematic Review. Value Health. 2024;27(4):527-41. Epub 20240129. doi: 10.1016/j.jval.2023.12.015. PubMed PMID: 38296049.

25. Manns B, Hemmelgarn B, Tonelli M, Au F, So H, Weaver R, et al. The Cost of Care for People With Chronic Kidney Disease. Can J Kidney Health Dis. 2019;6:2054358119835521. Epub 20190404. doi: 10.1177/2054358119835521. PubMed PMID: 31057803; PubMed Central PMCID: PMCPMC6452586.

26. Khadadah M. The cost of asthma in Kuwait. Med Princ Pract. 2013;22(1):87-91. Epub 20120808. doi: 10.1159/000341154. PubMed PMID: 22889866; PubMed Central PMCID: PMCPMC5586966.

27. Ruggeri M, Drago C, Mandolini D, Francesa Morel P, Mencacci C, Starace F. The costs of treatment resistant depression: evidence from a survey among Italian patients. Expert Rev Pharmacoecon Outcomes Res. 2022;22(3):437-44. Epub 20210726. doi: 10.1080/14737167.2021.1954507. PubMed PMID: 34240678.

28. Lastuka A, Bliss E, Breshock MR, Iannucci VC, Sogge W, Taylor KV, et al. Societal Costs of Dementia: 204 Countries, 2000-2019. J Alzheimers Dis. 2024;101(1):277-92. doi: 10.3233/jad-240163. PubMed PMID: 39150827; PubMed Central PMCID: PMCPMC11380273.

29. Kiltz U, Perez-Ruiz F, Uhlig T, Jansen TL, Karra R, Schmedt N, et al. Epidemiology, Treatment, and Health Resource Use of Gout Patients in Germany: Results from Analysis of a Claims Database. Value in Health. 2018;21:S7. doi: 10.1016/j.jval.2018.09.042.

30. Berni TR, Morgan CL, Rees DA. Rising Incidence, Health Resource Utilization, and Costs of Polycystic Ovary Syndrome in the United Kingdom. J Clin Endocrinol Metab. 2025;110(5):e1580-e9. doi: 10.1210/clinem/dgae518. PubMed PMID: 39049776; PubMed Central PMCID: PMCPMC12012766.
