# Supplementary material for: A comprehensive economic assessment of the burden of obesity in Kuwait
Source: PLoS One. 2026 Mar 4;21(3):e0344040. doi: 10.1371/journal.pone.0344040 (PMC12959657; doi:10.1371/journal.pone.0344040)
Supplement: S2 Table — (DOCX) [file pone.0344040.s002.docx]

**A comprehensive economic assessment of the burden of obesity in Kuwait**

**Supporting Information**

**S2 Table.** **Annual cost inputs per case (person)**

| **Disease** | **Cost in original study (Ref)** | **Country/ currency** | **Year of estimation** | **Inflated cost (KWD) 2024** | **Inflated cost (US$) 2024** | **Adjusted cost (PPP) 2024** |
| --- | --- | --- | --- | --- | --- | --- |
| **1. Direct medical costs** | | | | | | |
| **Esophagus adenocarcinoma** | 43,516 [1] | USA/US$ | 2020 | 16,144 | 52,755 | 52,755 |
| **Gastric cancer** | 46,501 [2] | Global/US$ | 2017 | 16,144 | 52,755 | 52,755 |
| **Colon and rectum cancers** | 58,384 [3] | Saudi Arabia/US$ | 2020 | 19,619 | 64,110 | 123,179 |
| **Liver cancer** | 53,954 [4] | Global/US$ | 2021 | 16,144 | 52,755 | 52,755 |
| **Pancreas cancer** | 40,357 [5] | European Union/Euro | 2019 | 16,169 | 52,837 | 68,682 |
| **Breast cancer** | 182,847 [6] | Saudi Arabia/SAR | 2018 | 16,597 | 54,236 | 104,209 |
| **Uterine/Endometrial cancer** | 43,516 [1] | USA/US$ | 2020 | 16,144 | 52,755 | 52,755 |
| **Ovary cancer** | 17,501 [7] | Spain/Euro | 2016 | 7,093 | 23,177 | 36,296 |
| **Prostate cancer** | 38,971 [8] | Switzerland/CHF | 2018 | 14,138 | 46,199 | 42,862 |
| **Kidney cancer** | 43,516 [1] | USA/US$ | 2020 | 16,144 | 52,755 | 52,755 |
| **Meningioma (Brain, central nervous system)** | 43,516 [1] | USA/US$ | 2020 | 16,144 | 52,755 | 52,755 |
| **Gallbladder cancer** | 43,516 [1] | USA/US$ | 2020 | 16,144 | 52,755 | 52,755 |
| **Thyroid cancer** | 43,516 [1] | USA/US$ | 2020 | 16,144 | 52,755 | 52,755 |
| **Non-Hodgkin lymphoma** | 43,516 [1] | USA/US$ | 2020 | 16,144 | 52,755 | 52,755 |
| **Multiple myeloma** | 43,516 [1] | USA/US$ | 2020 | 16,144 | 52,755 | 52,755 |
| **Leukemia** | 43,516 [1] | USA/US$ | 2020 | 16,144 | 52,755 | 52,755 |
| **Type II Diabetes mellitus** | 6,506 [9] | France/Euro | 2013 | 2,648 | 8,654 | 12,092 |
| **Hypertensive heart disease** | 7,287 [10] | Saudi Arabia/SAR | 2022 | 618 | 2,021 | 3,882 |
| **Ischaemic heart disease** | 10,064 [11] | Global/Int$ | 2019 | 3,780 | 12,353 | 12,353 |
| **Stroke** | 27,702 [12] | HICs/US$ | 2020 | 10,277 | 33,584 | 33,584 |
| **Atrial fibrillation and flutter** | 20,735 [13] | Global/Euro | 2022 | 7,380 | 24,115 | 31,347 |
| **Chronic kidney disease** | 14,634 [14] | Canada/Can$ | 2017 | 4,104 | 13,411 | 15,694 |
| **Asthma** | 406 [15] | Kuwait/US$ | 2012 | 538 | 1,757 | 2,701 |
| **Gastroesophageal reflux disease** | 30,000 [10] | Saudi Arabia/SAR | 2022 | 2,546 | 8,319 | 15,984 |
| **Nonalcoholic fatty liver** | 14,217 [10] | Saudi Arabia/SAR | 2022 | 1,206 | 3,942 | 7,575 |
| **Gallbladder diseases** | 14,217 [10] | Saudi Arabia/SAR | 2022 | 1,206 | 3,942 | 7,575 |
| **Depressive disorders** | 2,653 [16] | Italy/Euro | 2019 | 1,033 | 3,374 | 5,197 |
| **Alzheimer's disease and other dementias** | 2,486 [17] | Global/US$ | 2019 | 934 | 3,051 | 3,051 |
| **Osteoarthritis** | 12,342 [10] | Saudi Arabia/SAR | 2022 | 1,047 | 3,423 | 6,576 |
| **Back pain** | 12,342 [10] | Saudi Arabia/SAR | 2022 | 1,047 | 3,423 | 6,576 |
| **Gout** | 5,193 [18] | Germany/Euro | 2016 | 2,186 | 7,144 | 9,286 |
| **Polycystic ovarian syndrome** | 1,447 [19] | UK/GBP | 2019 | 696 | 2,274 | 2,741 |
| **Bariatric surgery** | 37,194 [10] | Saudi Arabia/SAR | 2022 | 3,156 | 10,314 | 19,818 |
| **2. Direct non-medical costs** | | | | | | |
| **Direct non-medical costs** | 8,658 [10] | Saudi Arabia/SAR | 2022 | 735 | 2,401 | 4,613 |
| **Out-of-pocket expenditures** | 1,177 [10] | Saudi Arabia/SAR | 2022 | 100 | 326 | 627 |
| **Cost of equipment and home renovation** | 365 [10] | Saudi Arabia/SAR | 2022 | 30 | 99 | 190 |
| **Cost of housekeeping** | 672 [10] | Saudi Arabia/SAR | 2022 | 57 | 186 | 358 |

*Can$: Canadian Dollar; CHF: Swiss Franc; GBP: Sterling Pound; Int$: International Dollar; KWD: Kuwaiti Dinar; PPP: Purchasing Power Parity; SAR: Saudi Arabian Rial; US$: United States dollars*
