# Supplementary material for: A comprehensive economic assessment of the burden of obesity in Kuwait
Source: PLoS One. 2026 Mar 4;21(3):e0344040. doi: 10.1371/journal.pone.0344040 (PMC12959657; doi:10.1371/journal.pone.0344040)
Supplement: S3 Table — (DOCX) [file pone.0344040.s003.docx]

**A comprehensive economic assessment of the burden of obesity in Kuwait**

**Supporting Information**

**S3 Table.** **Morbidity cases, mortality cases, costs (KWD), and YPLL attributable to obesity in Kuwait (per disease group)**

| **Disease group** | **Attributable cases** | **Direct medical cost** | **Direct non-medical cost** | **Indirect morbidity cost** | **Attributable deaths** | **Indirect mortality cost** | **YPLL** |
| --- | --- | --- | --- | --- | --- | --- | --- |
| **Type II Diabetes mellitus** | 458,859 | 1,215,223,930 | - | - | 167 | 17,869,327 | 3,066 |
| **Cardiovascular diseases** | 77,180 | 331,304,125 | - | - | 552 | 62,656,623 | 11,576 |
| **Gastrointestinal tract diseases** | 1,079,363 | 1,551,931,099 | - | - | 11 | 1,247,815 | 221 |
| **Musculoskeletal disorders** | 278,474 | 296,812,552 | - | - | - | - | - |
| **Cancers** | 4,606 | 77,329,431 | - | - | 109 | 14,402,164 | 3,019 |
| **Chronic kidney diseases** | 86,003 | 352,962,948 | - | - | 81 | 9,004,488 | 1,671 |
| **Asthma** | 17,948 | 9,647,491 | - | - | 4 | 407,779 | 73 |
| **Neuro/Mental conditions** | 30,421 | 31,287,027 | - | - | 37 | 3,157,700 | 437 |
| **Poly cystic ovarian syndrome** | 34,456 | 23,972,761 | - | - | - | - | - |
| **Obesity** | - | 31,100,409 | 37,947,123 | 225,445,828 | - | - | - |
| **TOTAL** | **2,067,311** | **3,921,571,775** | **37,947,123** | **225,445,828** | **960** | **108,745,896** | **20,062** |

*Kuwaiti Dinar; YPLL: years of potential life lost*
