# Supplementary material for: A comprehensive economic assessment of the burden of obesity in Kuwait
Source: PLoS One. 2026 Mar 4;21(3):e0344040. doi: 10.1371/journal.pone.0344040 (PMC12959657; doi:10.1371/journal.pone.0344040)
Supplement: Attachment — Submitted filename: Response to Reviewers.docx [file pone.0344040.s005.docx]

PONE-D-25-47395

A comprehensive economic assessment of the burden of obesity in Kuwait

Thank you for submitting your manuscript to PLOS ONE. After careful consideration, we feel that it has merit but does not fully meet PLOS ONE’s publication criteria as it currently stands. Therefore, we invite you to submit a revised version of the manuscript that addresses the points raised during the review process.

Please submit your revised manuscript by Mar 04 2026 11:59PM. If you will need more time than this to complete your revisions, please reply to this message or contact the journal office at plosone@plos.org. When you're ready to submit your revision, log on to https://www.editorialmanager.com/pone/ and select the 'Submissions Needing Revision' folder to locate your manuscript file.

Author response:

Date Sent:	23-Jan-2026

Dear Editor,

We are thrilled to have the opportunity to revise our manuscript and would like to express our gratitude to you and the reviewers for providing valuable comments and suggestions. These insights have been incredibly helpful in improving our manuscript. As a result, we have uploaded a revised copy of the manuscript with all the changes made during the revision process highlighted in blue.

We wanted to take a moment to show our appreciation for the time and effort you put into providing such insightful guidance. Our hope is that these revisions will elevate the paper and make it worthy of publication in PLOS ONE Journal. Herein, we offer detailed responses to all the comments and suggestions.

Thank you once again for your help and support.

Sincerely,

Corresponding author

- **Comment: Please include the following items when submitting your revised manuscript:**
- A letter that responds to each point raised by the academic editor and reviewer(s). You should upload this letter as a separate file labeled 'Response to Reviewers'.
- A marked-up copy of your manuscript that highlights changes made to the original version. You should upload this as a separate file labeled 'Revised Manuscript with Track Changes'.
- An unmarked version of your revised paper without tracked changes. You should upload this as a separate file labeled 'Manuscript'.
  - **Response:** We thank the editorial team for these clarifications.
  - **Action:** The items have been included as instructed.

- **Comment: If you would like to make** **changes to your financial disclosure, please include your updated statement in your cover letter. Guidelines for resubmitting your figure files are available below the reviewer comments at the end of this letter.**
  - **Response:** We thank the editorial team for this offer.
  - **Action:** No action has been taken as we have no changes to our financial disclosure.
- **Comment: If applicable, we recommend that you deposit your laboratory protocols in protocols.io to enhance the reproducibility of your results.**
  - **Response:** We thank the editorial team for this recommendation.
  - **Action:** No action has been taken as this recommendation is not applicable to our manuscript.

**Journal Requirements**:

- **Comment:** When submitting your revision, we need you to address these additional requirements.

**1. Please ensure that** **your manuscript meets PLOS ONE's style requirements, including those for file naming.**

- **Response:** We thank the editorial team for this clarification.
- **Action:** We updated the style of our manuscript to meet PLOS ONE's style requirements, including those for file naming.

**2. Please** **include** **captions for your Supporting Information files at the end of your manuscript, and update any in-text citations to match accordingly.**

- **Response:** We thank the editorial team for this recommendation.
- **Action:** We included captions for our Supporting Information files at the end of our manuscript, and update in-text citations to match accordingly.

**3. If the reviewer comments include a recommendation to cite specific previously published works, please review and evaluate these publications to determine whether they are relevant and should be cited.** There is no requirement to cite these works unless the editor has indicated otherwise.

- **Response:** We thank the editorial team for this clarification.
- **Action:** No action has been taken as this recommendation is not applicable to our manuscript.

**Reviewer's Responses to Questions**

**Comments to the Author**

1. Is the manuscript technically sound, and do the data support the conclusions?

The manuscript must describe a technically sound piece of scientific research with data that supports the conclusions. Experiments must have been conducted rigorously, with appropriate controls, replication, and sample sizes. The conclusions must be drawn appropriately based on the data presented.

Reviewer #1: Yes

- **Response:** We wanted to take a moment to show our appreciation for the time and effort you put into reviewing this manuscript.

2. Has the statistical analysis been performed appropriately and rigorously?

Reviewer #1: Yes

- **Response:** We wanted to take a moment to show our appreciation for the time and effort you put into reviewing this manuscript.

3. Have the authors made all data underlying the findings in their manuscript fully available?

The PLOS Data policy requires authors to make all data underlying the findings described in their manuscript fully available without restriction, with rare exception (please refer to the Data Availability Statement in the manuscript PDF file). The data should be provided as part of the manuscript or its supporting information, or deposited to a public repository. For example, in addition to summary statistics, the data points behind means, medians and variance measures should be available. If there are restrictions on publicly sharing data—e.g. participant privacy or use of data from a third party—those must be specified.

Reviewer #1: Yes

- **Response:** We wanted to take a moment to show our appreciation for the time and effort you put into reviewing this manuscript.

4. Is the manuscript presented in an intelligible fashion and written in standard English?

PLOS ONE does not copyedit accepted manuscripts, so the language in submitted articles must be clear, correct, and unambiguous. Any typographical or grammatical errors should be corrected at revision, so please note any specific errors here.

Reviewer #1: Yes

- **Response:** We wanted to take a moment to show our appreciation for the time and effort you put into reviewing this manuscript.

5. Review Comments to the Author

Please use the space provided to explain your answers to the questions above. You may also include additional comments for the author, including concerns about dual publication, research ethics, or publication ethics. (Please upload your review as an attachment if it exceeds 20,000 characters)

Reviewer #1: Title and Abstract,
The title accurately reflects the manuscript's focus on assessing the comprehensive economic burden of obesity in Kuwait. The abstract is well-structured, covering the study’s background, methodology, results, and conclusion concisely.
I suggest the following comments to improve the manuscript.
We wanted to take a moment to show our appreciation for the time and effort you put into reviewing this manuscript. We have addressed all comments, which we agree have further enhanced the paper's clarity and policy relevance.

- **Comment: Introduction:
  1. Add information about the economic burden** (e.g, medical direct cost, non-medical direct costs, and indirect costs) **of obesity in Kuwait. Through statistics, the mortality rate and comparison with other countries should be reported. Through statistics, the mortality rate, economic burden, and comparison with other countries should be reported.**
  - **Response:** We thank the reviewer for this very helpful framing suggestion.
  - **Action:** The text has been revised accordingly. Sentences have been added to provide this context and clarify the economic burden of obesity in Kuwait and the mortality rate and comparison with other countries.
  - **Revised Text (Line 93-100):** "This analysis used epidemiological and cost data from published studies and global datasets. It estimated Kuwait's 2019 burden at US$2.3 billion—1.7% of the country’s GDP—with projections rising to US$33.3 billion (9.42% of the country’s GDP) by 2060 [3]. In 2019, this compromised direct medical costs (US$638 million), direct non-medical costs (US$360 million), and indirect costs (US$1,660 million). Relative to other countries, Kuwait's obesity-related economic burden ranks among the highest, exceeding many high-income countries (e.g., 1.30% of the national GDP in Korea, 1.53% in Qatar, and 0.96% in Singapore) [3]. Moreover, Kuwait also ranks among the highest countries in terms of mortality attributable to high BMI globally. In 2023, high BMI accounted for 21.77% of all deaths in Kuwait, compared with 8.06% in high-income countries and 6.12% worldwide [14]. Complementing these findings, a retrospective micro-costing study from the public payer perspective focused on ten key obesity-related comorbidities."
- **Comment:**

**2. I invite the authors to write** **a summary of the** **importance of COI analysis in health care, and in measuring medical and other costs.**

- **Response:** We appreciate the reviewer’s insightful comment regarding the importance of COI analysis in health care.
- **Action:** We have added a summary as a new paragraph in the Introduction section to address the importance of cost-of-illness (COI) analysis in healthcare and its role in measuring medical and other costs.
- **Revised Text (Line 115-125):** " In this context, cost-of-illness (COI) studies offer a comprehensive approach, representing a key form of health economic analysis that quantifies the economic burden of diseases or risk factors like obesity on individuals and society. These analyses delineate direct medical costs (e.g., hospitalizations, pharmaceuticals), direct non-medical costs (e.g., transportation, accommodation), and indirect costs (e.g., productivity losses from morbidity and premature mortality), yielding reliable country-specific estimates vital for evaluating preventive or control interventions [16]**.** By informing resource allocation, prioritizing policies, raising awareness of obesity's societal impact, and justifying targeted budget measures—particularly in regions where such expenditures rival major health threats—COI studies underpin evidence-based decision-making and strategies extending beyond clinical outcomes [17]**.**"
- **Comment: Methods:**
  **3. To follow this section better, I invite the authors to write this section according to the EQUATOR network and related** **checklist/guideline.**
  URL: https://www.equator-network.org/
  - **Response:** We thank the reviewer for this invitation to enhance the Methods section’s structure and alignment with EQUATOR Network guidelines. While no specific reporting guideline exists for cost-of-illness (COI) studies within the EQUATOR Network, our manuscript already adheres to the consensus-based checklist for critical appraisal of COI studies, which promotes methodological rigor, transparency, and international comparability [**Schnitzler L, Roberts TE, Jackson LJ, Paulus ATG and Evers SMAA. A consensus-based checklist for the critical appraisal of cost-of-illness (COI) studies. *Int J Technol Assess Health Care*. 2023;39(1):e34. doi:10.1017/S026646232300019**].
  - **Action:** We have added the following sentence to contextualize the adherence to the consensus-based checklist for critical appraisal of COI studies developed by Schnitzler et al. (2023).
  - **Revised Text (Line 148-153):** " Importantly, to ensure methodological rigor, reporting quality, and international comparability, this study followed the consensus-based checklist for critical appraisal of COI studies developed by Schnitzler et al. (2023) [21]. This checklist comprises 17 main questions (plus sub questions) across three domains: study characteristics, methodology and cost analysis, and results and reporting. Notably, it has been adopted in several published COI studies [22-23].
- **Comment: Discussion**

**Policy implication section is missing (please add** **before limitation section)**

- **Response:** We appreciate the reviewer’s emphasis on the importance of including policy implication section to enhance the relevance and usefulness of this manuscript. A comprehensive policy implication section already exists in the Discussion part, outlining culturally tailored public health strategies, early detection programs, workplace interventions, digital tools, and expanded access to obesity treatments.
- **Action:** To enhance its prominence and address this comment, we have added a clear subheading—"Policy implications"—immediately before the Limitations section.

**Revised Text (Line: 519):** " Policy implications."

6. PLOS authors have the option to publish the peer review history of their article (what does this mean?). If published, this will include your full peer review and any attached files.

If you choose “no”, your identity will remain anonymous but your review may still be made public.

**Do you want your identity to be public for this peer review?** For information about this choice, including consent withdrawal, please see our Privacy Policy.

Reviewer #1: No

[NOTE: If reviewer comments were submitted as an attachment file, they will be attached to this email and accessible via the submission site. Please log into your account, locate the manuscript record, and check for the action link "View Attachments". If this link does not appear, there are no attachment files.]

- **Comment:** To ensure your figures meet our technical requirements, please review our figure guidelines: https://journals.plos.org/plosone/s/figures

You may also use PLOS’s free figure tool, NAAS, to help you prepare publication quality figures: https://journals.plos.org/plosone/s/figures#loc-tools-for-figure-preparation.

NAAS will assess whether your figures meet our technical requirements by comparing each figure against our figure specifications.

- **Response:** We thank the editorial team for this recommendation.
- **Action:** No action has been taken as this recommendation is not applicable to our manuscript.
