# Supplementary material for: A comprehensive economic assessment of the burden of obesity in Kuwait
Source: PLoS One. 2026 Mar 4;21(3):e0344040. doi: 10.1371/journal.pone.0344040 (PMC12959657; doi:10.1371/journal.pone.0344040)
Supplement: Attachment — Submitted filename: Response_to_Reviewers_auresp_2.docx [file pone.0344040.s006.docx]

PONE-D-25-47395R1

A comprehensive economic assessment of the burden of obesity in Kuwait

PLOS One

Dear Dr. Nagi,

Thank you for submitting your manuscript to PLOS ONE. After careful consideration, we feel that it has merit but does not fully meet PLOS ONE’s publication criteria as it currently stands. Therefore, we invite you to submit a revised version of the manuscript that addresses the points raised during the

Please submit your revised manuscript by Mar 28 2026 11:59PM. If you will need more time than this to complete your revisions, please reply to this message or contact the journal office at [plosone@plos.org](mailto:plosone@plos.org). When you're ready to submit your revision, log on to <https://www.editorialmanager.com/pone/> and select the 'Submissions Needing Revision' folder to locate your manuscript file.

Author response:

Date Sent:	11-Feb-2026

Dear Editor,

We are thrilled to have the opportunity to revise our manuscript and would like to express our gratitude to you and the reviewers for providing valuable comments and suggestions. These insights have been incredibly helpful in improving our manuscript. As a result, we have uploaded a revised copy of the manuscript with all the changes made during the revision process highlighted in blue.

We wanted to take a moment to show our appreciation for the time and effort you put into providing such insightful guidance. Our hope is that these revisions will elevate the paper and make it worthy of publication in PLOS ONE Journal. Herein, we offer detailed responses to all the comments and suggestions.

Thank you once again for your help and support.

Sincerely,

Corresponding author

- **Comment: Please include the following items when submitting your revised manuscript:**
- A letter that responds to each point raised by the academic editor and reviewer(s). You should upload this letter as a separate file labeled 'Response to Reviewers'.
- A marked-up copy of your manuscript that highlights changes made to the original version. You should upload this as a separate file labeled 'Revised Manuscript with Track Changes'.
- An unmarked version of your revised paper without tracked changes. You should upload this as a separate file labeled 'Manuscript'.
  - **Response:** We thank the editorial team for these clarifications.
  - **Action:** The items have been included as instructed.

- **Comment: If you would like to make** **changes to your financial disclosure, please include your updated statement in your cover letter. Guidelines for resubmitting your figure files are available below the reviewer comments at the end of this letter.**
  - **Response:** We thank the editorial team for this offer.
  - **Action:** No action has been taken as we have no changes to our financial disclosure.
- **Comment: If applicable, we recommend that you deposit your laboratory protocols in protocols.io to enhance the reproducibility of your results.**
  - **Response:** We thank the editorial team for this recommendation.
  - **Action:** No action has been taken as this recommendation is not applicable to our manuscript.

**Journal Requirements**:

- **Comment:** When submitting your revision, we need you to address these additional requirements.

**1. If the reviewer comments include a recommendation to cite specific previously published works, please review and evaluate these publications to determine whether they are relevant and should be cited.** There is no requirement to cite these works unless the editor has indicated otherwise.

- **Response:** We thank the editorial team for this clarification.
- **Action:** No action has been taken as this recommendation is not applicable to our manuscript.

**2. Please review y****our reference list to ensure that it is complete and correct.** If you have cited papers that have been retracted, please include the rationale for doing so in the manuscript text, or remove these references and replace them with relevant current references. Any changes to the reference list should be mentioned in the rebuttal letter that accompanies your revised manuscript. If you need to cite a retracted article, indicate the article’s retracted status in the References list and also include a citation and full reference for the retraction notice.

- **Response:** We thank the editorial team for this recommendation.
- **Action:** We reviewed our reference list and ensured it is complete and correct.

**Reviewers' comments:**

**Reviewer's Responses to Questions**

**Comments to the Author**
1. If the authors have adequately addressed your comments raised in a previous round of review and you feel that this manuscript is now acceptable for publication, you may indicate that here to bypass the “Comments to the Author” section, enter your conflict of interest statement in the “Confidential to Editor” section, and submit your "Accept" recommendation.

Reviewer #1: (No Response)

- **Response:** We have no response.

2. Is the manuscript technically sound, and do the data support the conclusions?

The manuscript must describe a technically sound piece of scientific research with data that supports the conclusions. Experiments must have been conducted rigorously, with appropriate controls, replication, and sample sizes. The conclusions must be drawn appropriately based on the data presented.

Reviewer #1: Yes

- **Response:** We wanted to take a moment to show our appreciation for the time and effort you put into reviewing this manuscript.

3. Has the statistical analysis been performed appropriately and rigorously?

Reviewer #1: Yes

- **Response:** We wanted to take a moment to show our appreciation for the time and effort you put into reviewing this manuscript.

4. Have the authors made all data underlying the findings in their manuscript fully available?

The PLOS Data policy requires authors to make all data underlying the findings described in their manuscript fully available without restriction, with rare exception (please refer to the Data Availability Statement in the manuscript PDF file). The data should be provided as part of the manuscript or its supporting information, or deposited to a public repository. For example, in addition to summary statistics, the data points behind means, medians and variance measures should be available. If there are restrictions on publicly sharing data—e.g. participant privacy or use of data from a third party—those must be specified.

Reviewer #1: Yes

- **Response:** We wanted to take a moment to show our appreciation for the time and effort you put into reviewing this manuscript.

5. Is the manuscript presented in an intelligible fashion and written in standard English?

PLOS ONE does not copyedit accepted manuscripts, so the language in submitted articles must be clear, correct, and unambiguous. Any typographical or grammatical errors should be corrected at revision, so please note any specific errors here.

Reviewer #1: Yes

- **Response:** We wanted to take a moment to show our appreciation for the time and effort you put into reviewing this manuscript.

6. Review Comments to the Author

Please use the space provided to explain your answers to the questions above. You may also include additional comments for the author, including concerns about dual publication, research ethics, or publication ethics. (Please upload your review as an attachment if it exceeds 20,000 characters)

Reviewer #1:

As a peer reviewer, I have carefully evaluated the submitted manuscript in terms of structure, scientific quality, methodology, and presentation.
Below are my detailed comments and recommendations for each section of the paper.
This paper estimates “The economic costs associated with obesity from governmental and societal perspectives in the State of Kuwait. The subject is of interest and very important in the field of Health Economics.
We wanted to take a moment to show our appreciation for the time and effort you put into reviewing this manuscript. We have addressed all comments, which we agree have further enhanced the paper's clarity.

- **Comment: Abstract:
  This section is enough.**
  - **Response:** We thank the reviewer for this feedback.
  - **Action:** No action has been taken.
- **Comment: Introduction**

**The introduction section is written well.**

- **Response:** We appreciate the reviewer’s encouraging statement.
- **Action:** No action has been taken.
- **Comment: Methods:**
  **Unit costs should be provided.**
  - **Response:** We thank the reviewer for this valuable recommendation. All unit costs are comprehensively detailed in S2 Table (Annual cost inputs per case) within the Supporting Information file, enhancing transparency in the Methods section.

**Hope this is satisfying**.

- **Comment: Results**

**This section is missing**

- **Response:** We thank the reviewer for highlighting this issue. Upon submission/resubmission, we confirm that the manuscript includes a comprehensive Results section (lines 329–415, pages 15–21), detailing all findings. This is further supplemented by S3 Table in the Supporting Information file. We apologize for any submission error that may have caused the section to appear missing and have verified its presence in the revised files.
- **Comment: Discussion**

**This section is written well. However, I invite the author compare the main results with those conducted in developed countries like the USA.**

- **Response:** We thank the reviewer for this constructive suggestion.
- **Action:** As suggested, we have incorporated findings from key US studies alongside previously cited high-income country comparisons (Portugal, Saudi Arabia, Italy, Switzerland) to strengthen the international benchmarking in the Discussion section (Line 434-448).
- **Revised Text (Line 444-446):** In contrast, earlier estimates reported direct medical costs comprising 43% of the total cost in the USA in 2019 [3] and 23.4% in 2023 [5].

7. PLOS authors have the option to publish the peer review history of their article (what does this mean?). If published, this will include your full peer review and any attached files.

If you choose “no”, your identity will remain anonymous but your review may still be made public.

**Do you want your identity to be public for this peer review?** For information about this choice, including consent withdrawal, please see our Privacy Policy.

Reviewer #1: No

[NOTE: If reviewer comments were submitted as an attachment file, they will be attached to this email and accessible via the submission site. Please log into your account, locate the manuscript record, and check for the action link "View Attachments". If this link does not appear, there are no attachment files.]

- **Comment:** To ensure your figures meet our technical requirements, please review our figure guidelines: https://journals.plos.org/plosone/s/figures

You may also use PLOS’s free figure tool, NAAS, to help you prepare publication quality figures: https://journals.plos.org/plosone/s/figures#loc-tools-for-figure-preparation.

NAAS will assess whether your figures meet our technical requirements by comparing each figure against our figure specifications.

- **Response:** We thank the editorial team for this recommendation.
- **Action:** No action has been taken as this recommendation is not applicable to our manuscript.
